# Conditional VHL Gene Deletion Causes Hypoglycemic Death Associated with Disproportionately Increased Glucose Uptake by Hepatocytes through an Upregulated IGF-I Receptor

**DOI:** 10.1371/journal.pone.0069139

**Published:** 2013-07-09

**Authors:** Atsushi Kurabayashi, Yoshihiko Kakinuma, Taku Morita, Keiji Inoue, Takayuki Sato, Mutsuo Furihata

**Affiliations:** 1 Department of Pathology, Kochi Medical School, Nankoku, Kochi, Japan; 2 Department of Cardiovascular Control, Kochi Medical School, Nankoku, Kochi, Japan; 3 Department of Pediatrics, Ashiya Municipal Hospital, Ashiya, Hyogo, Japan; 4 Department of Urology, Kochi Medical School, Nankoku, Kochi, Japan; University of Otago, New Zealand

## Abstract

Our conditional *VHL* knockout (*VHL-KO*) mice, having *VHL* gene deletion induced by tamoxifen, developed severe hypoglycemia associated with disproportionately increased storage of PAS-positive substances in the liver and resulted in the death of these mice. This hypoglycemic state was neither due to impaired insulin secretion nor insulin receptor hypersensitivity. By focusing on insulin-like growth factor I (IGF-I), which has a similar effect on glucose metabolism as the insulin receptor, we demonstrated that IGF-I receptor (IGF-IR) protein expression in the liver was upregulated in *VHL-KO* mice compared to that in the mice without *VHL* deletion, as was the expression of glucose transporter (GLUT) 1. The interaction of the receptor for activated C kinase (RACK) 1, which predominantly binds to VHL, was enhanced in *VHL-KO* livers with IGF-IR, because *VHL* deletion increased free RACK1 and facilitated the IGF-IR-RACKI interaction. An IGF-IR antagonist retarded hypoglycemic progression and sustained an euglycemic state. These IGF-IR antagonist effects on restoring blood glucose levels also attenuated PAS-positive substance storage in the liver. Because the effect of IGF-I on HIF-1α protein synthesis is mediated by IGF-IR, our results indicated that *VHL* inactivation accelerated hepatic glucose storage through the upregulation of IGF-IR and GLUT1 and that IGF-IR was a key regulator in *VHL*-deficient hepatocytes.

## Introduction

The von Hippel-Lindau (*VHL*) gene is a tumor suppressor. Thus, *VHL* malfunctions predispose to clear-cell renal cell carcinoma (ccRCC) [1]. The hypoxia-inducible factor (HIF) system plays a major role in protecting cells against hypoxic insults and its protein levels are regulated by VHL protein (pVHL) [2] through the ubiquitin-mediated degradation of HIF [2]–[4].

In hypoxia, disrupted interactions between HIF-1α and VHL causes more HIF protein to escape degradation. As a result, HIF-1α upregulates the expressions of downstream genes, such as vascular endothelial cell growth factor (VEGF) and glucose transporter (GLUT) 1 and 3.

To date, our research has focused on the effects of the HIF system on organ protection. First, using an *in vivo* Cre-lox P system, conditional *VHL* knockout (*VHL-KO*) mice have demonstrated that renal tubular injury induced by ischemia-reperfusion injury was attenuated by deleting the *VHL* gene [5]. Second, conditional *VHL* knockdown appropriately activated the nitric oxide (NO)-VEGF axis to salvage glomerular endothelial cells from glomerulonephropathy induced by Habu snake venom [6]. VEGF activates NO production by increasing endothelial NOS (eNOS) expression and this balanced activation of the VEGF-NO pathway induces a survival signal in endothelial cells that maintains their function [7]. In addition, a previous study reported that eNOS deficiency resulted in insulin resistance in mice [8]. Taken together, it is possible that NO produced along with VEGF in a proportionate manner is an important factor involved in cell protection as well as in glucose utilization for glucose homeostasis.

The insulin-like growth factor I (IGF-I) receptor (IGF-IR) is known to mediate various cellular processes [9]–[12]. Insulin and IGF-I exert their biological effects through the insulin receptor (IR) and the IGF-IR, respectively, which share heterodimeric α_2_β_2_ structures [13]. The IR and IGF-IR can bind to each other’s ligands, but with different affinities [14], [15]. Because the insulin receptor (IR) and IGF-IR contributed distinct signals to common downstream components in response to each of their ligands [16], IGF-I could mimic insulin’s effects on glucose metabolism by acting through IGF-IR [17].

The receptor for activated C kinase 1 (RACK1) is the first member to be identified in the RACK family [18]. RACK1 may have a pivotal role in many critical biological responses by acting as a mediator that integrates different signaling pathways [19]. Previous studies demonstrated that IGF-I-treated pVHL-deficient RCC cells exhibited increased IGF-IR-RACK1 interactions and had increased IGF-IR and Akt activation [20], [21]. This suggested that pVHL competed with the IGF-IR for RACK1 binding and played another critical role in glucose metabolism in a HIF system-independent manner [20], [21].

With regard to glucose metabolism mediated by the HIF system, two studies have reported that *VHL* disruption in adult hepatocytes resulted in increased glycogen granules, lipid droplets, and premature death due to hypoglycemia within weeks [22], [23]. Downregulating the GLUT2 and glucose-6-phosphatase (G-6-Pase) genes by *VHL* deletion impedes the appropriate release of glucose from the liver, which results in abnormal hepatic accumulation of glycogen and hypoglycemia in *VHL*-deficient mice [22]. It was also reported that the HIF system inhibited mitochondrial respiration, impaired fatty acid oxidation, and reduced ketone and glucose production [23].

In this study, we demonstrated that *VHL*-inactivated hepatocytes had enhanced IGF-IR protein expression concomitant with enhanced IGF-IR and RACK1 interactions and glucose uptake in the liver, which resulted in severe hypoglycemia. These results identified a novel role for *VHL* in hepatic glucose utilization.

## Materials and Methods

### Production of VHL Conditional Knockout (*VHL-KO*) Mice

All animal procedures were approved by the Animal Research Committee of Kochi Medical School (Permit Number: F-00072) and performed in strict accordance with guidelines of this committee. All surgery was performed under sodium pentobarbital anesthesia, and all efforts were made to minimize suffering. Mice harboring the floxed *VHL* allele were produced by Ma *et al.* using Cre/lox site-specific recombination technology [24], in which a tamoxifen-inducible Cre recombinase transgene was driven by a human β-actin promoter (*VHL^f/d^CreER™*) [5], [6]. To obtain an adequate number of mice for experiments, each *VHL^f/d^CreER™* mouse with a C57BL6/J genetic background was further crossed to produce both *VHL^f/f^CreER™* and *VHL^f/d^CreER™* mice, i.e., *VHL-KO* mice. One week (or nine days) before the experiments, mice were injected i.p. with tamoxifen (Sigma-Aldrich, St. Louis, MO, USA) in corn oil (4 mg/mouse) to express Cre recombinase. Male and female (8–16 weeks old) *VHL^f/d^CreER™* or *VHL^f/f^CreER™* mice, demonstrated as control mice without induction, were subjected to tamoxifen induction for experiments, and they were used as *VHL-KO* mice. On the other hand, *VHL^f/+^* mice, and C57BL6/J wild-type mice were also subjected to induction as negative control.

To monitor the *VHL-KO* specific phenotype hypoglycemia, blood samples were collected from the tail of conscious mice and blood glucose concentrations were measured with a glucometer (Accu-chek Active II, Roche) at least once per week. Mice were sacrificed quickly by cervical dislocation to minimize suffering, and liver with other organs were excised from mice for further experiments. Moreover, to administer pharmacological reagents including an IGF-IR antagonist, a mouse anesthetized with sodium pentobarbital was loaded subcutaneously on the back with an osmotic pump.

### Cell Culture

Cells of liver cancer cell line Huh-7 were cultured in DMEM (WAKO, Osaka, Japan) supplemented with 10% heat-inactivated fetal bovine serum (FBS), 10000 U/mL of penicillin G, 10000 µg/mL of streptomycin sulfate, and 25 µg/mL of amphotericin B. Cells were cultured as adherent cells in a humidified atmosphere at 37°C in 5% CO_2_.

### Small Interfering RNA Transfection

In this study, validated VHL small interfering RNA (siRNA) with the following sequence was used: sense: 5′-UCUCUCAAUGUUGACGGACAGCCUA-3′, antisense: 5′-UAGGCUGUCCGUCAACAUUGAGAGA-3′ (Life Technologies, Inc., Rockville, MD, USA). Huh-7 cells that were grown in 24-well plates were transfected with 50 nM of siRNA using Lipofectamine RNAi MAX and Opti-MEM medium (Life Technologies) according to the manufacturer’s recommendations. Negative control siRNA was also obtained from Life Technologies.

### Preparation of Protein Extracts and Immunoprecipitation

Protein extracts from whole livers were prepared using standard procedures for Western blot analysis and immunoprecipitation (IP) using a Universal Magnetic Co-IP Kit (Carlsbad, CA, USA) according to the manufacturer’s protocol. Protein concentration was determined by BCA protein assay (Thermo Scientific, Rockford, IL, USA). Total protein (500 µg) was incubated with a rabbit polyclonal anti-IGF-I receptor β (IGF-IR) antibody (1∶100; Cell Signaling Technology, Danvers, MA, USA) or rabbit polyclonal anti-Insulin receptor β (IR) antibody (1∶50; Santa Cruz Biotechnology, Inc., Santa Cruz, CA, USA) with gentle rocking at 4°C for 2 or 4 h. Protein G magnetic beads were used to precipitate IGF-IR or IR complexes by incubating for 1 h at 4°C. Samples were centrifuged, re-suspended in loading buffer (130 mM Tris pH 6.8, 4% SDS, 0.02% bromophenol blue, 20% glycerol, 100 mM DTT), boiled for 10 min, and subjected to Western blot analysis.

### Western Blot Analysis

Western blot analysis was performed as described previously [5], [6]. Protein extracts were electrophoresed and transferred to polyvinylidene difluoride membranes (Immobilon-P; Millipore, Bedford, MA, USA). The membranes were probed with the following antibodies: mouse monoclonal anti-RACK1 antibody (1∶1000; BD Biosciences, San Jose, CA, USA); rabbit polyclonal anti-IGF-IR antibody (1∶500; Cell Signaling Technology); rabbit polyclonal anti-IR antibody (1∶200; Santa Cruz Biotechnology); mouse monoclonal anti-VHL antibody (1∶100; BD Biosciences); mouse monoclonal anti-HIF-Iα antibody (1∶500; Novus Biologicals, Littleton, CO, USA); mouse monoclonal anti-Phospho-Akt antibody (Ser473) (1∶500; Cell Signaling Technology); rabbit polyclonal anti-GLUT1 antibody (1∶2000; Millipore, Bedford, MA, USA); rabbit polyclonal anti-GLUT2 antibody (1∶2000; Millipore); rabbit polyclonal anti-GLUT3 (1∶4000; Abcam Inc, Cambridge, MA, USA); and rabbit polyclonal anti-GLUT4 antibody (1∶2000; Millipore). These antibodies were used in conjunction with a horseradish peroxidase-conjugated secondary antibody. Comparable sample loading volumes were confirmed by expression of mouse monoclonal anti-β-actin antibody (1∶5000; Sigma-Aldrich).

### Intraperitoneal Glucose Tolerance Test and Acute Insulin Secretory Response in vivo

After fasting for 16–18 h, *VHL^f/d^CreER™* and *VHL^f/f^CreER™ (VHL-KO)* mice were weighed and blood glucose levels were determined using an Accu-check glucometer (Roche Diagnostics, Basel, Switzerland). Mice were injected intraperitoneally with glucose at 10 µL/g body weight, and blood glucose levels were sequentially determined before and 30 and 60 min after glucose injection. Serum insulin concentrations were determined using an Ultra Sensitive Mouse Insulin ELISA Kit (Morinaga Institute of Biological Science, Inc., Yokohama, Japan) according to the manufacturer’s instructions. *VHL^f/d^CreER™* and *VHL^f/f^CreER™* mice without tamoxifen injection and C57BL6/J wild type with/without tamoxifen injection were used as comparison.

### Histopathology and Immunohistochemical Analysis

Pancreatic and hepatic tissues were processed for routine paraffin-wax histology and sections were stained with hematoxylin and eosin (H&E). Liver tissues were also evaluated by Periodic and acid/Schiff (PAS) staining, PAS diastase (PAS-D) staining, and oil-red O staining. For pancreatic histology, five separate sections were examined to measure the diameters of the Islets of Langerhans. Immunohistochemical analysis used a Ventana automated immunohistochemistry system (Discovery TM; Ventana Medical Systems, Inc., Tucson, AZ, USA). The following primary antibodies were used for histological examinations: guinea pig anti-insulin antibody (1∶400; Dako cytomation, Glostrup, Denmark); rabbit polyclonal anti-glucagon antibody (1∶50; Abcam); rabbit polyclonal anti-VHL antibody (1∶20; Santa Cruz Biotechnology); and rabbit polyclonal anti-IGF-IR antibody (1∶500; Cell Signaling Technology). Primary antibodies were detected with biotinylated anti-rabbit IgG (1∶50; Dako Cytomation).

### STZ Treatment

Mice were administered i.p. injections of streptozotocin (STZ) (Sigma-Aldrich, St. Louis, MO, USA) at 100 mg/kg per day for 2 consecutive days. STZ was freshly dissolved in 0.01 M citrate buffer (pH 4.5) prior to each injection. One week after the first STZ injection, STZ-induced diabetic mice were obtained. In this study, two groups were treated with STZ injection. In one group, *VHL-KO* mice were developed by initial treatment with i.p. injection of STZ, followed by induction. In another group, mice were treated with tamoxifen for *VHL* deletion, followed by STZ injection a week later.

### _L_-NAME-treated Mice

*VHL-KO* mice were treated with *N*_ω_-Nitro-L-arginine methyl ester hydrochloride (_L_-NAME, Sigma-Aldrich) using osmotic pumps (DURECT Corporation, Cupertino, CA, USA) as described previously [25]. The osmotic pumps were implanted subcutaneously, which provided for a constant systemic administration (62.5 µg/µL/h) of _L_-NAME during the experiment (14 days). *VHL-KO* mice treated with 0.9% NaCl were used as controls. Two days after pump implantation, mice were injected with tamoxifen. Non-fasting blood glucose levels (BS) were determined before (BS_before_) and seven days after (BS_after_) the tamoxifen injection. Data were used to determine ΔBS values: ΔBS = BS_after_ – BS_before_.

### eNOS-deficient Mice

Homozygous eNOS^−/−^ mice (The Jackson Laboratory, Bar harbor, ME, USA) were intercrossed with *VHL-KO* mice and heterozygous mice (*VHL^+/f^CreER^TM^eNOS^+/−^*) were mated with each other to obtain mice that lacked both the *eNOS* and *VHL* (*VHL^f/f^CreER^TM^eNOS^−/−^*) genes. These mice were injected with tamoxifen to actively express Cre recombinase. ΔBS values were determined as with _L_-NAME-treated mice.

### IGF-IR Antagonist-treated Mice

To identify a key molecule responsible for the hypoglycemic state observed in *VHL-KO* mice, we examined the blood glucose levels in *VHL-KO* mice after they were treated with an IGF-IR inhibitor. *VHL-KO* mice were treated for 14 days using osmotic pumps (0.25 µg/µL/h 25% of acetic acid) with either an IGF-IR antagonist (Bachem, Torrance, CA, USA) or a linear IGF-IR antagonist (Operon, Tokyo, Japan), which had different protein structures but had identical amino acid sequences, and therefore, could not bind to IGF-IR. *VHL^f/+^* mice that were treated with an IGF-IR antagonist and *VHL-KO* mice that were treated with 25% acetic acid were used as controls. Two days after pump implantation, the mice were injected with tamoxifen. After this experiment, the mouse livers were excised and examined by H&E staining and PAS staining.

### Glucagon Assays

Serum glucagon levels were determined using a YK090 Glucagon EIA kit (Yanaihara Institute, Inc., Shizuoka, Japan).

### 2-NBDG Determinations

*VHL^f/f^CreER™* mice, with/without tamoxifen injection, were anesthetized with pentobarbital. Then, 2-NBDG [2-(N-(7-nitrobenz-2-oxa-1,3-diazol-4-yl)amino)-2-deoxyglucose] dissolved in 0.9% NaCl was injected via the internal jugular vein (250 µg/mouse). The livers were quickly harvested 2 h after the 2-NBDG injection and analyzed for 2-NBDG on frozen sections. Liver 2-NBDG fluorescent images were examined using a fluorescence microscope (Olympus, Tokyo, Japan) with excitation at 490 nm and emission at 530 nm.

### Statistical Analysis

Results are given as means ± standard errors of the mean (SEM). Paired t-tests and Mann–Whitney U tests were used to compare two groups. P-values of <0.05 were considered statistically significant.

## Results

### Glucose Metabolism and Pancreatic β cell Function in *VHL-KO* Mice

**Figure 1:**
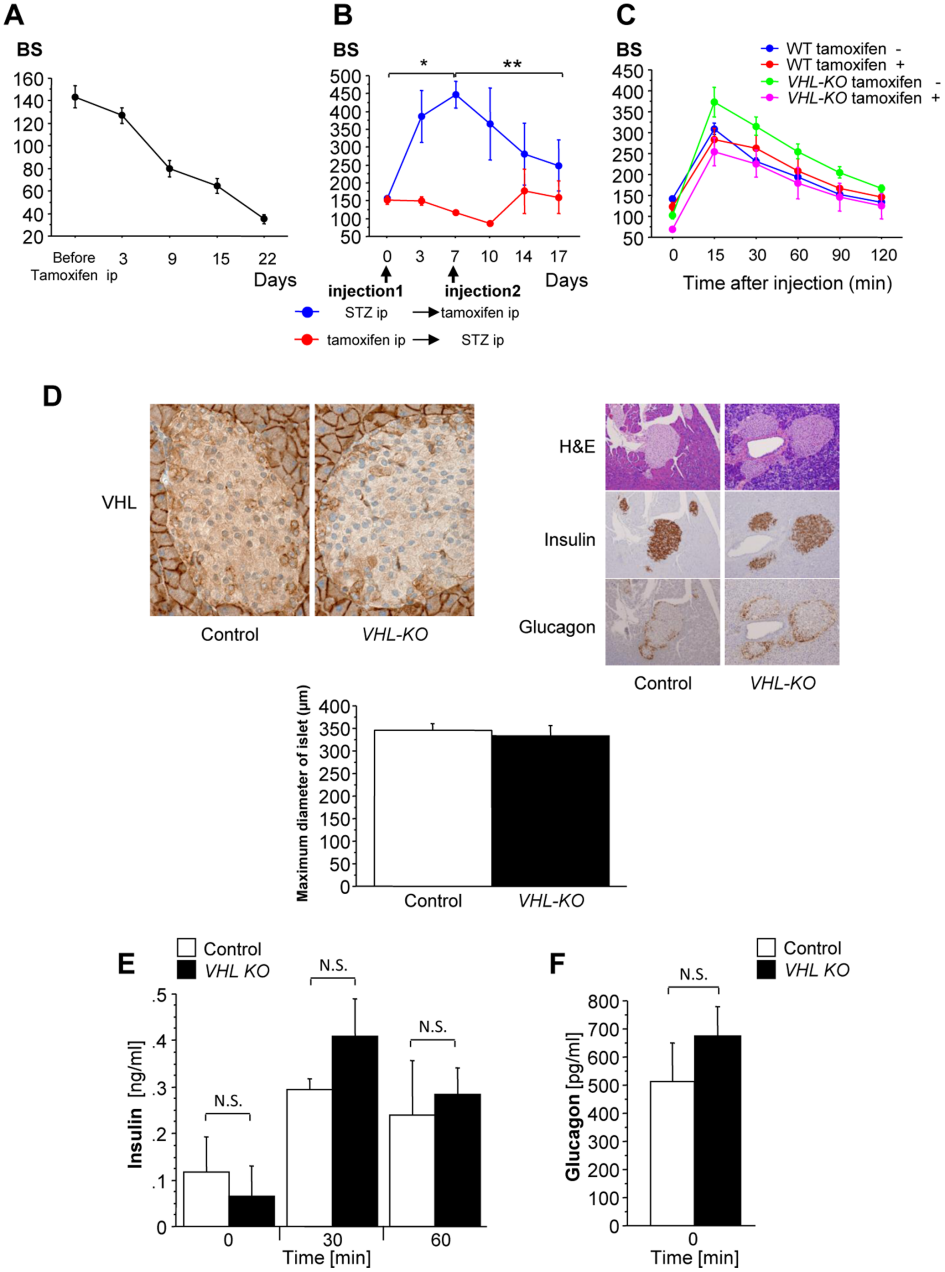
*VHL-KO* mice exhibit hypoglycemia despite normal glucose tolerance and intact pancreatic β cells. (**A**) *VHL-KO* mice had significant decreases in blood glucose levels (BS) after tamoxifen injection (4 mg/mouse; n = 10). (B) *VHL-KO* mice were treated with streptozotocin (STZ) before or after *VHL-*knockdown (n = 4 per group). Before tamoxifen injection, STZ treated mice (blue line) had significant increases in BS compared with their pre-STZ-blood glucose levels. After tamoxifen injection, their BS gradually decreased (day 0 vs. day 7, *p = 0.0057; day 7 vs. day 17, **p = 0.036). The mice treated with STZ after tamoxifen injection (red line) did not show any significant increases in blood glucose levels throughout the experiment. (**C**) *VHL-KO* mice (*VHL-KO* tamoxifen +) had glucose tolerance results comparable to that of other mice (WT tamoxifen +/− and *VHL-KO* tamoxifen −): WT tamoxifen −, n = 11; WT tamoxifen +, n = 10; *VHL-KO* tamoxifen −, n = 11; *VHL-KO* tamoxifen +, n = 7. (**D**) Histopathological images of pancreatic tissues showed that the immunoreactivity patterns for insulin and glucagon and morphological changes were not affected by *VHL* deletion with comparable maximum diameters of islets between control (*VHL-KO* tamoxifen −) and *VHL-KO* mice. (**E**) Glucose tolerance tests showed similar trends in insulin concentrations between *VHL-KO* and control mice (n = 5 per group, Time 0 min, *p* = 0.773:N.S.; 30 min, *p* = 0.294: N.S.; 60 min, *p*>0.9999: N.S.). (**F**) Basal glucagon levels were comparable between *VHL*-*KO* and control mice (*p* = 0.465: N.S.).

*VHL^f/d^CreER™* (*VHL-KO*) mice had significant decreases in their blood glucose levels during the follow-up period after tamoxifen injection (Figure 1A). To investigate whether the hypoglycemia in *VHL-KO* mice was insulin-dependent, *VHL-KO* mice were treated with streptozotocin (STZ) to develop insulin-deficient diabetes.

Prior to *VHL* deletion, STZ significantly increased blood glucose levels compared with non-treated mice (*p = *0.0057). However, after this deletion, blood glucose levels continued to decrease (*p = *0.036) and finally declined to the hypoglycemic level. In contrast, the mice treated with STZ after *VHL-KO* did not show any significant increases in blood glucose levels throughout the experiment (Figure 1B), which suggested that hypoglycemia may not have been due to an insulin-dependent effect.

In the glucose tolerance test, the blood glucose levels in C57BL6/J with/without tamoxifen and *VHL^f/d^CreER™* mice with/without tamoxifen revealed no significant differences during the follow-up period (Figure 1C). Histopathological images of pancreatic tissues, particularly islets of Langerhans, showed that there were no morphological changes or immunohistological changes in insulin and glucagon distributions between control and *VHL-KO* mice, while the VHL expression level decreased in *VHL-KO* mice, compared to control mice (Figure 1D, top panel). The diameters of the islets of Langerhans (maximum diameters) were not significantly different between control and *VHL-KO* mice (Figure 1D, bottom graph).

In the fasted state, basal insulin levels were comparable between the *VHL-KO* (*VHL^f/f^CreER™* with tamoxifen) and control (*VHL^f/f^CreER™* without tamoxifen) mice (Figure 1E, *p = *0.773: not significantly different (N.S.) in Time 0 min,). Furthermore, glucose challenge increased serum insulin concentrations to comparable levels in both *VHL-KO* and control mice (Figure 1E, Time 30 min, *p = *0.294: N.S.; Time 60 min, *p*>0.9999: N.S). In addition, basal glucagon levels were similar between *VHL*-*KO* and control mice (Figure 1F, *p = *0.465: N.S.). These results also strengthened our speculation that because *VHL-KO* mice had normal glucose tolerance with normal β cell function, the hypoglycemia observed in *VHL-KO* mice may be independent of an insulin signaling cascade.

### Glucose Metabolism in _L_-NAME-treated *VHL-KO* Mice and eNOS Deficient *VHL-KO* Mice

**Figure 2:**
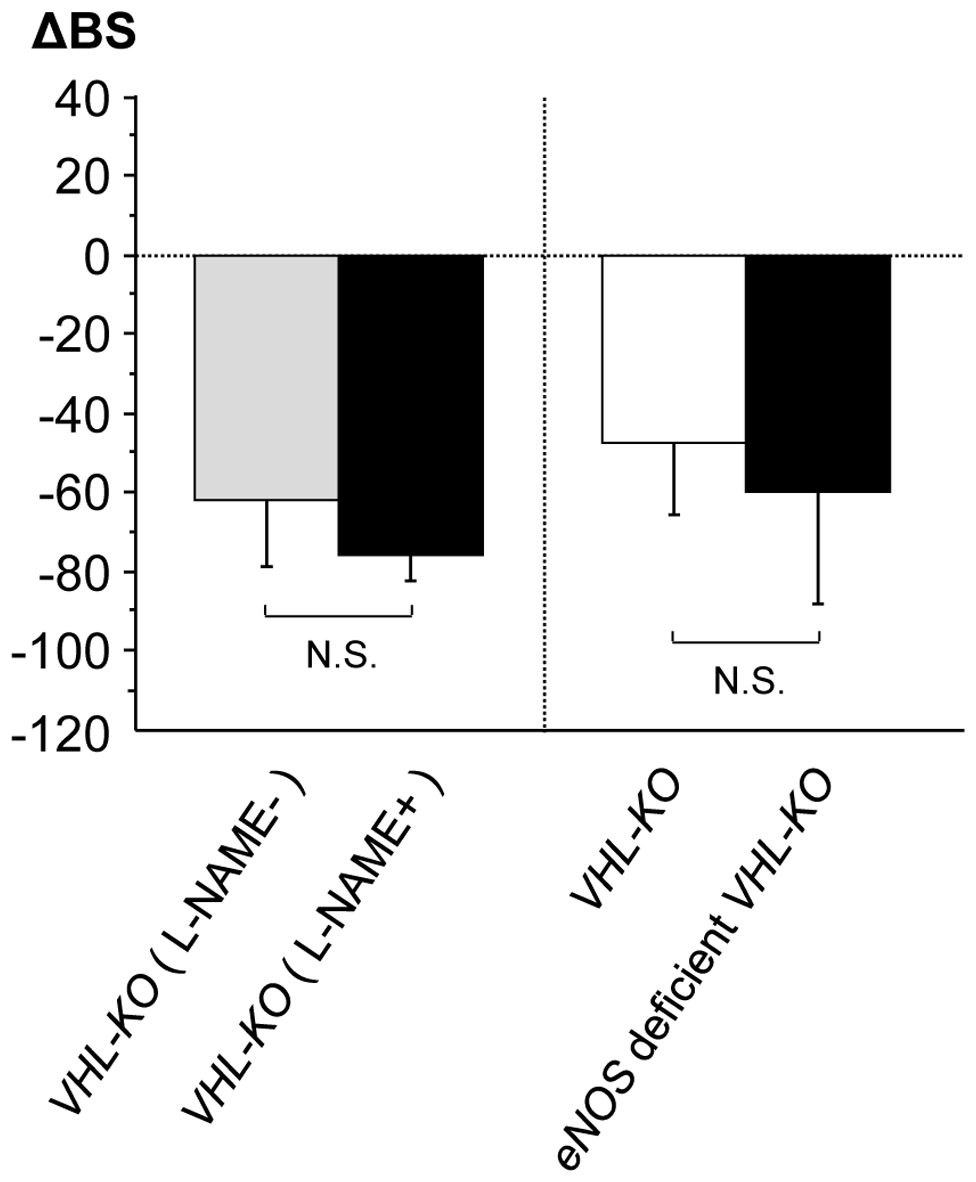
NO production due to *VHL* deletion does not contribute to hypoglycemia in *VHL-KO* mice. There were no significant increases in blood glucose levels (BS) in both _L_-NAME-treated *VHL-KO* mice (n = 5) and *eNOS*-deficient *VHL*-*KO* mice (n = 5). The hypoglycemic levels of both these mouse groups were not different from those of *VHL-KO* mice (n = 6–7) (_L_-NAME-treated *VHL-KO* mice vs. *VHL-KO* mice, *p* = 0.210: N.S.; *eNOS*-deficient *VHL*-*KO* mice vs. *VHL-KO* mice, *p* = 0.523: N.S.). ΔBS was determined by subtracting BS before the tamoxifen injection from BS after the injection. Data were used to determine ΔBS values: ΔBS = BS_after_ – BS_before_.

To confirm that insulin sensitivity was not mediated by increased NO in *VHL-KO* mice, we measured blood glucose levels in _L_-NAME-treated *VHL-KO* mice and eNOS deficient *VHL-KO* mice. As shown in Figure 2, there were no differences in the reduced blood glucose levels (i.e., hypoglycemic levels) between _L_-NAME-treated *VHL-KO* and untreated *VHL-KO* mice (*p = *0.210: N.S.). In addition, there were no differences in hypoglycemic severity between eNOS-deficient *VHL-KO* (*VHL^f/f^CreER^TM^eNOS^−/−^* with tamoxifen) and *VHL-KO* (*VHL^f/f^CreER™* with tamoxifen) mice (*p = *0.523: N.S.). Although our recent study demonstrated that enhanced NO production in glomeruli prevented the kidney from endothelial cell-targeted nephropathy, the results here implied that upregulated NO production induced by *VHL* deletion did not contribute to hypoglycemia in *VHL-KO* mice.

### Histological Changes and Glucose Metabolism in the Liver after *VHL*-deletion

**Figure 3:**
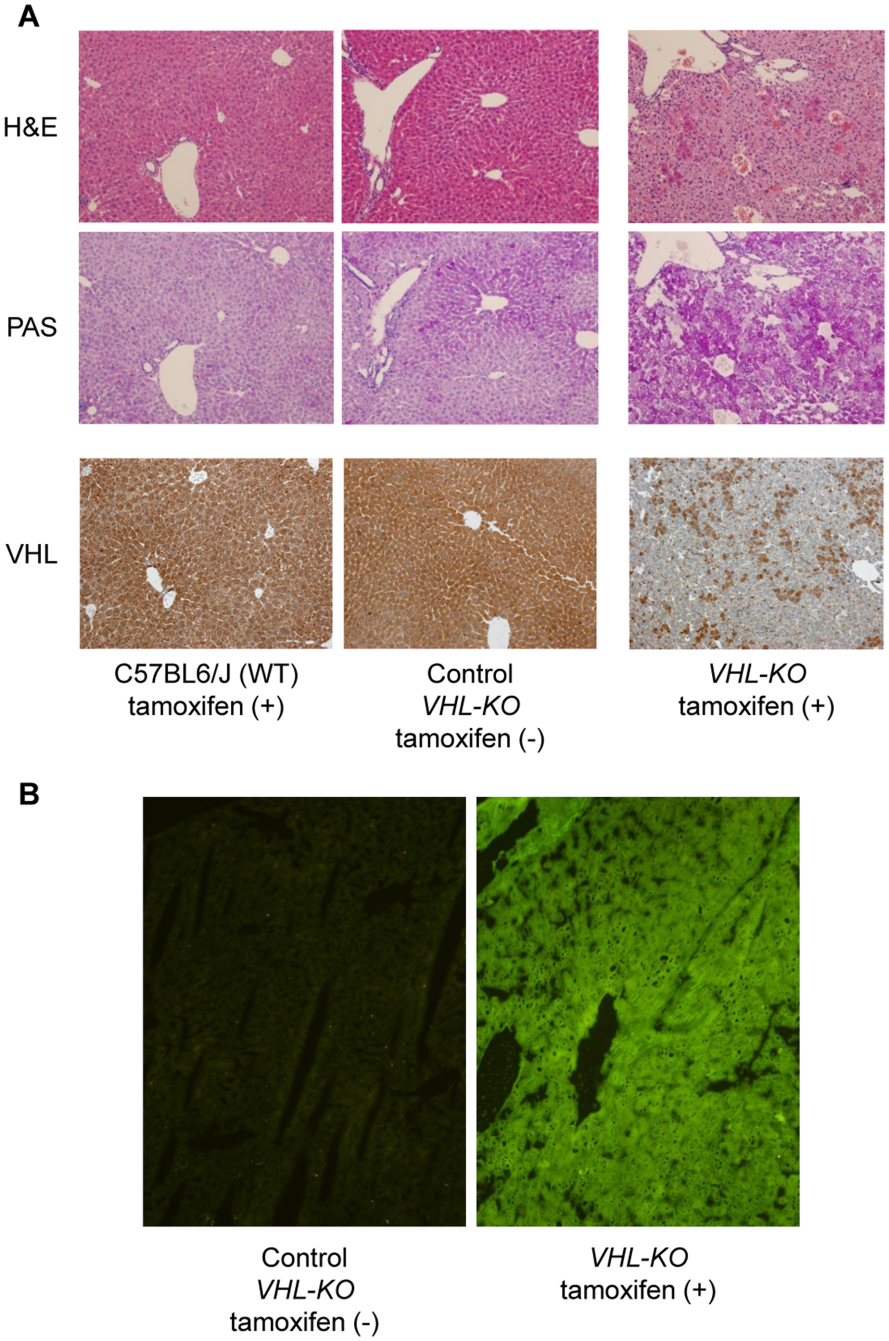
*VHL* deletion induces accelerated hepatic glucose uptake and storage of PAS-positive substances. (**A**) H&E stained liver sections from *VHL-KO* mice showed characteristic appearances of hepatocytes (top panel); translucent hepatocytes with conserved cellular architectures. These translucent hepatocytes were strongly positive for PAS staining (middle panel) compared to those cells from WT Tamoxifen+and *VHL-KO* Tamoxifen −, which suggested marked glycogen accumulation. VHL immunostaining showed mosaic loss of VHL expression in the liver in *VHL-KO* mice (bottom panel). (**B**) In *VHL-KO* mice, 2-NBDG fluorescence intensity in the liver was considerably increased compared to control mice (*VHL-KO* Tamoxifen −).

Histological examinations of hematoxylin and eosin (H&E) stained *VHL-KO* liver sections revealed that the cytoplasm of adult hepatocytes turned translucent with a clear appearance (Figure 3A, top panel). In previous studies, accumulation of fat within hepatocytes was confirmed with oil-red O staining in another type of *VHL-KO* mouse [22], [23]. We also confirmed fat accumulation using this method (data not shown).

We also examined whether there were alterations in glycogen metabolism in the *VHL-KO* liver, as a lack of H&E staining in the hepatocyte cytoplasm often involves glycogen accumulation [26]. These translucent hepatocytes were strongly positive for Periodic and acid/Schiff (PAS) staining (Figure 3A, middle panel). In contrast, they were negative for PAS diastase (PAS-D) staining, which suggested marked glycogen accumulation in the *VHL-KO* liver (data not shown). We checked VHL deletion in the liver in *VHL-KO* mice by immunohistochemistry, and confirmed mosaic loss of VHL expression in the liver in *VHL-KO* mice (Figure 3A, bottom panel). In the livers of *VHL-KO* (*VHL^f/f^CreER™* with tamoxifen) mice, the fluorescence level of a glucose analogue, 2-NBDG [2-(N-(7-nitrobenz-2-oxa-1,3-diazol-4-yl)amino)-2-deoxyglucose], which was injected intravenously, was much higher than that in the livers of control (*VHL^f/f^CreER™* without tamoxifen) mice (Figure 3B). This result indicated that glucose uptake into hepatocytes was enhanced by *VHL* deletion. The fluorescence intensity in the liver of *VHL-KO* mice was the highest among the organs examined, including skeletal muscle and heart, although, compared with the levels in control mice, the fluorescence levels in the skeletal muscle and heart of *VHL-KO* mice were markedly higher (data not shown).

### *In vivo* Association of IGF-IR with RACK-I in the Liver with *VHL*-deletion

**Figure 4:**
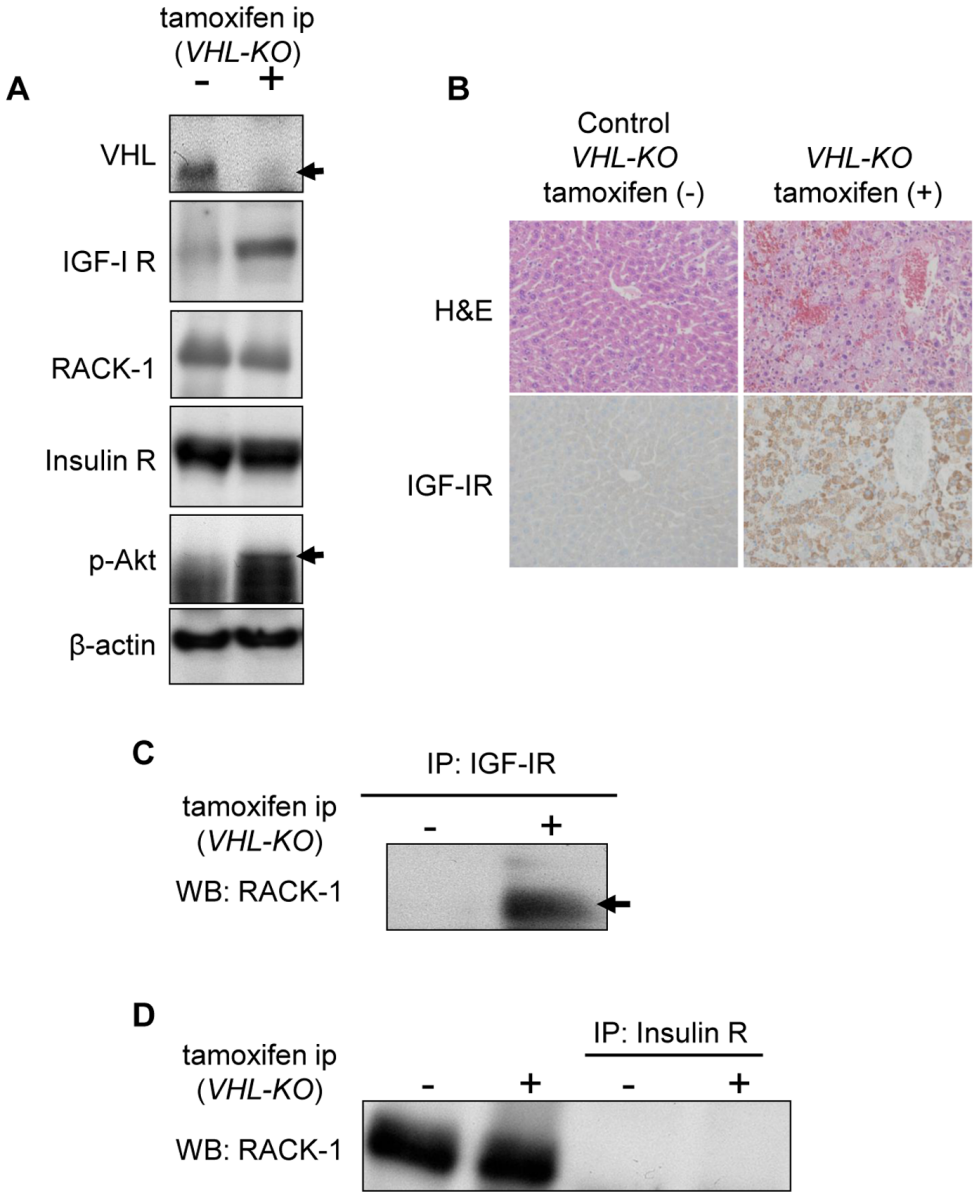
IGF-IR expression and IGF-IR interaction with RACK1 are upregulated in *VHL-KO* livers. (**A**) *VHL-KO* livers resulted in downregulation of VHL expression (top panel). *VHL-KO* livers had significantly higher levels of IGF-IR compared to control livers. p-Akt expression was also enhanced in *VHL-KO* livers. No significant effects of *VHL* deletion were observed for the expression levels of RACK1 and IR. (**B**) IGF-IR immunoreactivity was increased in *VHL-KO* livers. (**C**) Immunoprecipitation (IP) of *VHL-KO* liver cell lysates using an anti-IGF-IR antibody were followed by immunoblotting with an anti-RACK1 antibody. In the *VHL-KO* liver lysates, the interaction between IGF-IR and RACK1 was markedly enhanced. (**D**) However, immunoprecipitated hepatocyte lysates from both *VHL-KO* and control mice using an anti-IR antibody did not contain RACK1.

The insulin-like growth factor I receptor (IGF-IR), receptor for activated C kinase 1 (RACK1), insulin receptor (IR), phospho-Akt (p-Akt) and VHL expression in the livers of *VHL-KO* (*VHL^f/f^CreER™* with tamoxifen) and control (*VHL^f/f^CreER™* without tamoxifen) mice were evaluated by Western blot analysis (Figure 4A). *VHL-KO* livers resulted in downregulation of VHL expression (Figure 4A, top panel). *VHL-KO* livers had significantly higher levels of IGF-IR compared to control livers, as also supported by immunohistochemical analysis (Figure 4B). Phospho-Akt expression was also enhanced in the livers of *VHL-KO* mice. On the other hand, RACK1 and IR expression levels in *VHL-KO* livers were comparable with those in control livers. To evaluate the interaction of IGF-IR with RACK1 in *VHL-KO* livers, we conducted co-immunoprecipitation (co-IP) experiments using liver cell lysates from *VHL-KO* and control mice. On using an anti-IGF-IR antibody, it was observed that immunoprecipitates from *VHL-KO* livers included more RACK1 protein compared with those from control mice (Figure 4C). In contrast, the IR immunoprecipitates from *VHL-KO* and control livers did not include RACK1 (Figure 4D). Taken together, IGF-IR formed a complex more efficiently with RACK1 in the *VHL-KO* liver than in the control liver.

### Association between *VHL*-deletion and IGF-IR Expression *in vitro*

**Figure 5:**
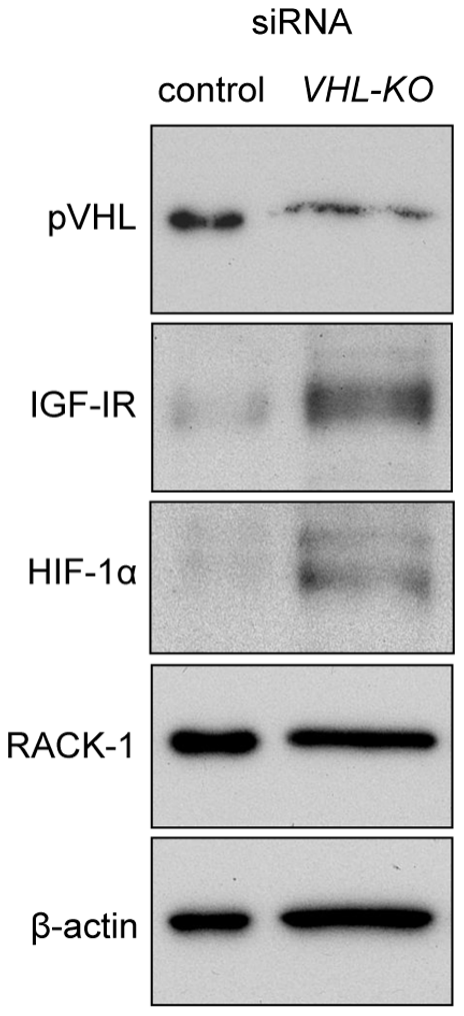
IGF-IR expression levels are increased in human liver Huh-7 cells by *VHL* deletion. Transfecting VHL siRNA into Huh-7 cells resulted in downregulation of VHL expression (top panel). Reciprocally, IGF-IR and HIF-Iα expressions levels were increased by *VHL-*deletion. No significant effects of *VHL* deletion were observed on the expression levels of RACK1.

VHL siRNA introduced into Huh-7 cells resulted in downregulation of VHL expression (Figure 5, top panel). In agreement with the *in vivo* experiments using *VHL-KO* mice, IGF-IR and HIF-Iα expression were enhanced by *VHL* knockdown, although RACK1 expression levels were comparable with those in control, which suggested that *VHL* knockdown directly led to IGF-IR upregulation.

### The Effects of IGF-IR Inhibition on Glucose Metabolism in *VHL-KO* Mice

**Figure 6:**
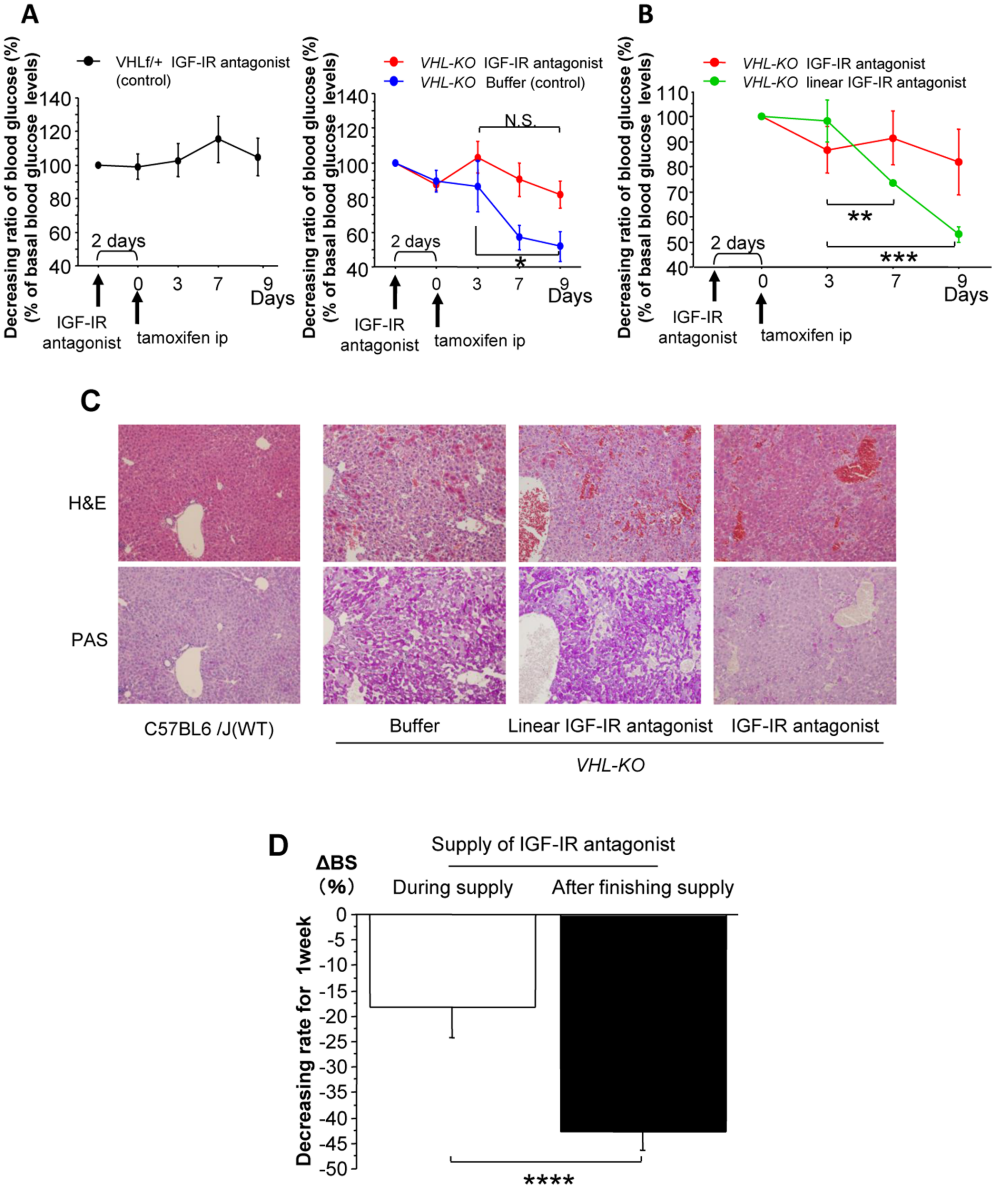
IGF-IR inhibition attenuates hypoglycemia. (**A**) An IGF-IR antagonist did not affect blood glucose levels in control mice (left panel, n = 3). Compared to buffer treated-*VHL-KO* control mice (blue line, n = 5; day3 vs. day9, *p = 0.040), administration of an IGF-IR antagonist (red line, n = 5) resulted in significant recovery from hypoglycemia (day 3 vs. day 9, *p* = 0.121: N.S.; right panel). (**B**) In contrast, the blood glucose levels in *VHL-KO* mice treated with a linear IGF-IR antagonist (green line, n = 5) were significantly decreased during the experiment, like those of buffer-treated mice (day 3 vs. day 7, **p = 0.037; day 3 vs. day 9, ***p = 0.0025). Linear IGF-IR antagonist had different protein structures but had identical amino acid sequences, and therefore, could not bind to IGF-IR. (**C**) For IGF-IR antagonist-treated *VHL-KO* mice, hepatic glycogen accumulation was attenuated compared to that in the livers of linear IGF-IR antagonist-treated and buffer-treated mice. (**D**) In IGF-IR antagonist-treated *VHL-KO* mice, glucose levels rapidly decreased after discontinuing the IGF-IR antagonist treatment (****p = 0.023).

As shown in Figure 6A, the IGF-IR inhibition did not modulate the blood glucose levels in control mice (Figure 6A, left panel). In contrast, compared to buffer treated-*VHL-KO* control mice (day 3 vs. day 9 glucose levels, *p = *0.040; Figure 6A, right panel), IGF-IR antagonist administration resulted in attenuation of hypoglycemia after tamoxifen injection (day 3 vs. day 9, *p = *0.121: N.S.). In contrast, a linear IGF-IR antagonist did not increase the blood glucose levels. In *VHL-KO* mice, the IGF-IR antagonist restored the blood glucose levels, whereas the linear IGF-IR antagonist did not (day 3 vs. day 7, *p = *0.037; day 3 vs. day 9, *p = *0.0025; Figure 6B).

These results were accompanied by an inhibitory effect of the IGF-IR antagonist on glycogen accumulation in *VHL-KO* mice (Figure 6C). After discontinuing the IGF-IR antagonist administration, the blood glucose levels in *VHL-KO* mice that had been maintained by the antagonist rapidly declined (*p = *0.023; Figure 6D). These results indicated that IGF-IR played an important role in glucose uptake and hypoglycemia in *VHL-KO* mice.

### *In vivo* Association between *VHL*-deletion and Glucose Transporter Expression in the Liver

**Figure 7:**
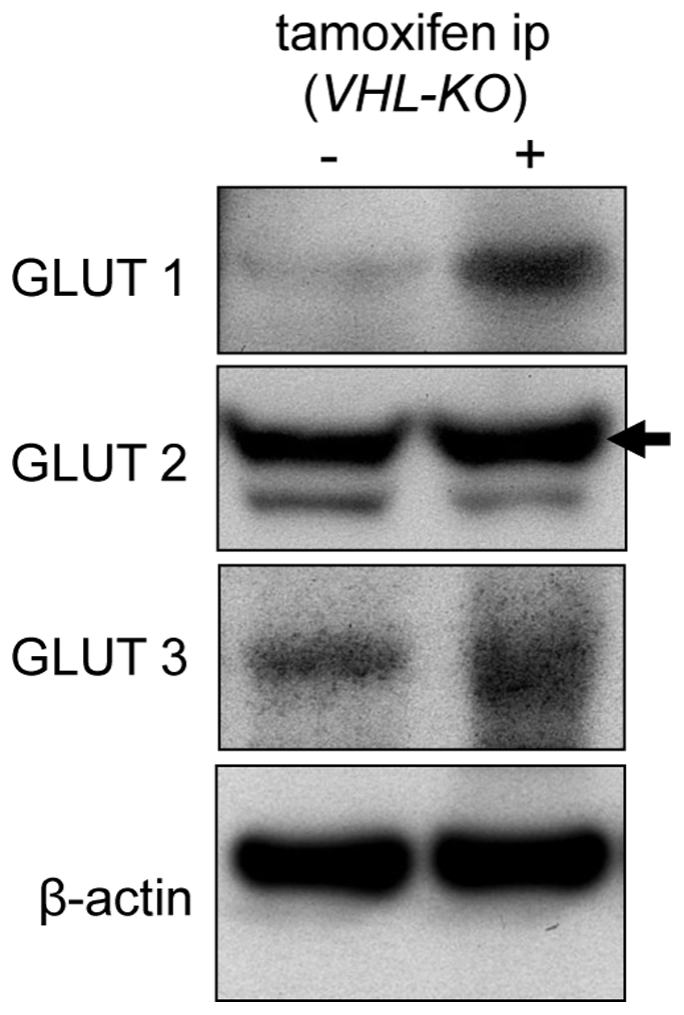
GLUT1 was markedly enhanced in *VHL-KO* livers. Expressions of GLUT1 (top panel) and GLUT3 (bottom panel), particularly GLUT1, are enhanced in *VHL-KO* livers. GLUT2 expression level in *VHL-KO* livers was comparable to that in the control livers (middle panel).

To determine the glucose transporters predominantly responsible for glucose uptake together with IGF-IR activation, the protein expressions of GLUT1, GLUT2, GLUT3, and GLUT4 were analyzed by Western blots. GLUT1 and GLUT3 expression, particularly that of GLUT1, was markedly enhanced in *VHL-KO* (*VHL^f/f^CreER™* with tamoxifen) livers, whereas that of GLUT2 was not (Figure 7). GLUT4 expression in *VHL-KO* livers was comparable to that in the control (*VHL^f/f^CreER™* without tamoxifen) livers (data not shown).

## Discussion

In the current study, we revealed that *VHL* deletion remarkably enhanced glucose uptake into hepatocytes and caused severe hypoglycemia, which resulted in the death of these mice. To gain further insights into the function of *VHL* in glucose metabolism, we performed extensive experiments using a *VHL-KO* mouse model based on the tamoxifen-inducible *CreER™* system to inactivate the *VHL* gene in organs in a time-specific manner.

Previous studies reported that mice that lacked *VHL* in pancreatic β cells had impaired glucose tolerance and that glucose stimulated insulin secretion was severely reduced [27], [28]. Cantley *et al*. also suggested that β cells that lacked *VHL* had abnormalities in glucose sensing and that the VHL/HIF pathway was critical for regulating mammalian pancreatic β cell function [28]. In contrast, quite distinct from these previous studies, the *VHL-KO* mouse model in our study retained glucose tolerance and had normal β cell function in terms of releasing adequate amounts of insulin in response to the glucose load.

As reported by Duplain *et al.*, NO has an accelerating effect on glucose uptake to enhance insulin sensitivity [8]. However, this was not the case with our *VHL-KO* mice, as both _L_-NAME and *eNOS* deletion in *VHL-KO* mice did not increase their blood glucose levels. Taken together, it was unlikely that the hypoglycemic state observed in *VHL-KO* mice resulted from impaired insulin secretion or insulin receptor sensitivity.

Among numerous studies using genetically modified *VHL*, only two studies have addressed the role of *VHL* in unregulated hepatic glycogen storage. Park *et al.* demonstrated that *VHL*-inactivation lead to abnormal hepatic glycogen accumulation and that downregulated GLUT2 and glucose-6-phosphatase (G-6-Pase) expression hindered efficient glucose release from the liver, which resulted in an unexpected accumulation of glycogen [22]. Kucejova *et al.* reported that HIF mediated suppression of mitochondrial respiration caused impaired fatty acid oxidation and reduced glucose production, which ultimately resulted in hypoglycemia and death [23]. However, these two studies did not identify any critical molecules that were responsible for the hypoglycemic phenotype, which may have been regulated through HIF.

However, we showed that 2-NBDG fluorescence intensity in the livers of *VHL-KO* mice was much higher than that in control mice due to enhanced uptake of 2-NBDG. 2-NBDG uptake was accelerated to a greater level in hepatocytes compared to that in the skeletal muscle and heart in *VHL-KO* mice. These results suggested that *VHL* deletion-induced enhancement of glucose uptake in the liver could be attributed to hypoglycemia.

Insulin and IGF-I can bind to each other’s receptors, although their binding affinity to the non-cognate receptor is 100-fold lower than that to their own cognate receptor [14], [15]. Di Cola *et al.* reported that IGF-I could mimic effects of insulin on glucose metabolism through its own receptor in IR deficient mice [17]. Yuen *et al.* reported that pVHL suppressed IGF-IR promoter activity through its interaction with Sp1, and also reduced the stability of *IGF-IR* mRNA via the sequestration of HuR [29]. Consequently, *VHL* inactivation would be expected to upregulate IGF-IR in RCC. In addition, He *et al.* reported that pVHL interacted with RACK1 to disrupt the association between RACK1 and IGF-IR, which suggested that RACK1 was a direct mediator connecting the loss of pVHL function with an enhanced IGF-IR/Akt/MMP-2 signaling pathway in RCC [20].

Consistent with these reports, our *VHL-KO* mice had enhanced IGF-IR expression in the liver and an enhanced interaction between IGF-IR and RACK1. In addition, p-Akt expression was also enhanced in *VHL-KO* livers. Based on the previous reports and our data, we postulated that hepatic *VHL* deletion activated an IGF-IR pathway through an accelerated complex formation with RACK1 and contributed to severe hypoglycemia. Indeed, administrating an IGF-IR antagonist resulted in complete suppression of hypoglycemic progression in *VHL-KO* mice. In addition to maintaining the blood glucose levels, hepatic histological changes (i.e., accumulation of PAS positive substances like glycogen) were also attenuated in *VHL-KO* mice. These results also strongly supported our hypothesis. The reciprocal changes between *VHL* deletion and IGF-IR upregulation were confirmed with an *in vitro* experiment using human liver Huh-7 cells, where *VHL* knockdown cells had reciprocally increased IGF-IR expression.

IGF-I induces the expressions of HIF-1α and HIF-1 targets (i.e., GLUT1 or VEGF) in human colon cancer cells [30] and rat cerebral cortex [31]. This was independent of hypoxia-induced inhibition of ubiquitination [30], as Chavez *et al.* reported that a neutralizing anti-IGF-I antibody did not affect hypoxia-induced HIF-1α accumulation [31]. Thus, IGF-I and hypoxia activate the HIF system through independent mechanisms. In addition, these studies reported that inhibiting IGF-IR abrogated HIF-1 accumulation, which demonstrated a requirement for signal transduction via IGF-IR.

In our previous study, the protein levels of HIF-1α and HIF-2α were increased in *VHL-KO* mice kidneys [5]. In addition, in this study, HIF-1α upregulation were confirmed with an *in vitro* experiment using human liver Huh-7 cells by *VHL* knockdown. Furthermore, in this study, IGF-IR expression was also increased in *VHL-KO* hepatocytes. The protein levels of GLUT1, one of the targets regulated by HIF-1α, were increased in *VHL-*inactivated hepatocytes; however, the GLUT 2 expression levels were comparable to those of the controls. Unexpectedly, inhibiting the IGF-IR signal with an IGF-IR antagonist sustained the blood glucose levels and remarkably suppressed the hepatic phenotypes. Therefore, the upregulated glucose transporters that were involved in *VHL-KO* mice were suspected to be GLUT1.

In summary, *VHL-KO* mice exhibited severe hypoglycemia. Along with the severe hypoglycemia and hepatic glycogen accumulation in the liver, hepatic IGF-IR expression was upregulated and the IGF-IR-RACK1 interaction was enhanced. Administering an IGF-IR antagonist resulted in significant improvements in the hypoglycemic state and glycogen accumulation. Our results indicated that IGF-IR was a key regulator of the increased glucose uptake into *VHL*-deficient hepatocytes. To our knowledge, this is the first study to show that *VHL-KO* mice have increased glucose uptake into hepatocytes via IGF-IR activation, which contributes to their severe hypoglycemia.
